# NTCP Deficiency Affects the Levels of Circulating Bile Acids and Induces Osteoporosis

**DOI:** 10.3389/fendo.2022.898750

**Published:** 2022-07-22

**Authors:** Fangji Yang, Wenxiong Xu, Lina Wu, Luo Yang, Shu Zhu, Lu Wang, Wenbin Wu, Yuzhen Zhang, Yutian Chong, Liang Peng

**Affiliations:** ^1^ Department of Infectious Diseases, Third Affiliated Hospital of Sun Yat-sen University, Guangzhou, China; ^2^ Department of Spine Surgery, Third Affiliated Hospital of Sun Yat-sen University, Guangzhou, China; ^3^ Key Laboratory of Liver Disease of Guangdong Province, Third Affiliated Hospital of Sun Yat-sen University, Guangzhou, China

**Keywords:** sodium taurocholate co-transporting polypeptide (NTCP), SLC10A1, bile acid, hypercholanemia, osteoporosis, vitamin D, mutation

## Abstract

**Background:**

The p.Ser267Phe mutation in the SLC10A1 gene can cause NTCP deficiency. However, the full clinical presentation of p.Ser267Phe homozygous individuals and its long-term consequences remain unclear. Hence, in the present study, we characterized the phenotypic characteristics of NTCP deficiency and evaluated its long-term prognosis.

**Methods:**

Ten NTCP p.Ser267Phe homozygous individuals were recruited and a comprehensive medical evaluation with a 5-year follow-up observation was performed. The phenotypic characteristics of NTCP deficiency were also demonstrated using an NTCP-global knockout mouse model.

**Results:**

During the 5-year follow-up observation of 10 NTCP p.Ser267Phe homozygous adults, we found that the most common phenotypic features of NTCP deficiency in adults were hypercholanemia, vitamin D deficiency, bone loss, and gallbladder abnormalities. The profile of bile acids (BAs) in the serum was significantly altered in these individuals and marked by both elevated proportion and concentration of primary and conjugated BAs. Moreover, the NTCP deficiency led to increased levels of serum BAs, decreased levels of vitamin D, and aggravated the osteoporotic phenotype induced by estrogen withdrawal in mice.

**Conclusions:**

Both mice and humans with NTCP deficiency presented hypercholanemia and were more prone to vitamin D deficiency and aggravated osteoporotic phenotype. Therefore, we recommend monitoring the levels of BAs and vitamin D, bone density, and abdominal ultrasounds in individuals with NTCP deficiency.

## Introduction

The sodium taurocholate co-transporting polypeptide (NTCP) is encoded by the solute carrier family 10 member 1 (*SLC10A1*) gene. NTCP is a bile acid (BA) transporter and a cellular receptor for hepatitis B virus (HBV) and hepatitis D virus (HDV) (1, 2). Moreover, BAs represent a major class of cholesterol-derived amphipathic molecules regulating cholesterol metabolism, promoting bile secretion, and facilitating the digestion and absorption of lipids (3). The homeostasis of BAs is maintained by the synthesis in the liver, canalicular secretion, microbial metabolism, and efficient intestinal reabsorption into the blood (4).

Furthermore, NTCP plays a central role in the enterohepatic circulation of BAs, uptaking BAs from portal blood into hepatocytes to maintain enterohepatic recirculation, which has been confirmed *in vitro* and *in vivo* (1, 3, 5, 6). The NTCP deficiency in humans and mice can lead to increased levels of BAs in the blood, thereby corroborating the important role of NTCP in the transport of conjugated BAs (7, 8). The administration of a selective NTCP-binding peptide used as an entry inhibitor for HBV and HDV, myrcludex B, results in elevated levels of conjugated bile salts in healthy volunteers and chronic HDV infection patients (9, 10). Additionally, some *SLC10A1* polymorphisms have been reported to render NTCP loss of function *in vitro* and *in vivo*. The genetic variants c.190G>A (Ala64Thr), c.668T>C (Ile223Thr), c.800C>T (Ser267Phe), and c.836T>C (Ile279Thr) have also been reported to impair BA transport activity *in vitro* (11–13). In 2015, the first case of NTCP deficiency due to a mutation was reported and presented massive elevation of BAs in the serum, growth retardation, and motor delay without pruritus or generalized jaundice (7). Then, several cases with deleterious *SLC10A1* mutations causing NTCP deficiency have been published and all NTCP-deficienct patients presented hypercholanemia characterized by elevated BA levels in the circulation (8). On the other hand, the NTCP deficiency caused by the p.Ser267Phe homozygous mutation in *SLC10A1* is mostly asymptomatic with persistent hypercholanemia (8, 14). However, in our previous study, carriers of the p.Ser267Phe homozygous mutation presented not only asymptomatic hypercholanemia, but also vitamin D deficiency, and altered levels of sex hormones and blood lipids (15).

Until now, the phenotypic characteristics of NTCP deficiency and its long-term prognosis have not been comprehensively described. Besides, data on the profile of elevated BAs and long-term prognosis caused by NTCP deficiency are also scarce. Therefore, in the present study, we characterized the phenotypic characteristics of NTCP deficiency in adults, described the profile of serum BA profile in patients with the NTCP p.Ser267Phe mutation, and evaluated its long-term prognosis in adults.

## Materials and Methods

### Subjects and Samples

This study included 10 homozygous (HOM) and 20 healthy heterozygous (HET) individuals with the p.Ser267Phe *SLC10A1* mutation, and 20 healthy wild-type (WT) age-matched volunteers. The recruitment of the cohort, except for three homozygous individuals, was described previously (15). Three subjects with higher levels of total BAs in the serum but without clear causes were recruited from clinics and *SLC10A1* sequencing was performed. Serum samples were obtained in the morning from patients in a fasted state. Homozygotic samples were collected twice within a 12- to 18-month interval. All participants signed the informed written consent and this study followed the Declaration of Helsinki and the project was approved by the Ethics Committee of The Third Affiliated Hospital of Sun Yat-sen University [(2015) 2-155].

### Generation of NTCP Knockout (KO) Mice Models

NTCP-KO mice lacking exon 2 were designed on the C57BL/6J background and generated by the Shanghai Model Organisms Center (Shanghai, China) using the CRISPR/Cas9 technology. Mice were housed and bred under a 12-hour light-dark cycle and received standard chow and water. After female mice grew to 8 weeks old, they were either sham or ovariectomized. The use of animals was approved by the Institutional Animal Care and Use Committee from South China Agricultural University[2021D078].

### Clinical Parameters and Serum Analyses of Volunteers and Mice

We analyzed different clinical parameters of the homozygous individuals recruited in this study, including the levels of total BAs in the serum, liver function, blood lipids, and vitamin (A, B2, B5, D, E, K1) levels in the serum or plasma (BGI Clinical Lab, China). Osteoprotegerin (OPG) and receptor activator of NF-κβ ligand (RANKL) in the serum of mice were measured using ELISA kits (Boster Biotechnology, Wuhan, China), according to the manufacturer’s instructions.

### Quantification and Profiling of BAs in the Serum

The high-throughput profiling of BAs was performed on a UPLC-MS/MS system (ACQUITY UPLC-Xevo TQ-S, Waters Corp., Milford, MA, USA). The comprehensive BA profiling and quantification were performed according to previously published protocols (16). Forty-one species of BAs were tested in human serum samples and 15 species of BAs were detected in serum samples of mice. The list of 41 BA standards and the abbreviation are supplied in the **Table S1**.

### Micro- Computed Tomography (CT) Analyses

The micro-CT imaging was performed using Siemens Inveon CT Scanner with a voxel size of 9 mm. Image reconstruction was carried out using Inveon Acquisition workplace software. The procedure was performed according to previously published protocols (17).

### Histology

First, mice femurs were fixed in formalin, embedded in paraffin, then sectioned. Next, hematoxylin-eosin (H&E) and tartrate-resistant acid phosphatase (TRAP) stainings were performed as previously described (17).

### Statistical Analyses

Data are provided as means ± standard deviations (SDs) or medians with quartiles (interquartile range, IQR). Differences between groups were analyzed using the Kruskal-Wallis test, Student’s t-tests, or analysis of variance (ANOVA). A two-tailed *p-*value < 0.05 was considered statistically significant. Graphs were generated using GraphPad Prism 6.0 (GraphPad Software Inc., USA) or R Programming Language.

## Results

### Clinical Features of Individuals Carrying the Homozygous p.Ser267Phe Mutation in NTCP

**Figure 1:**
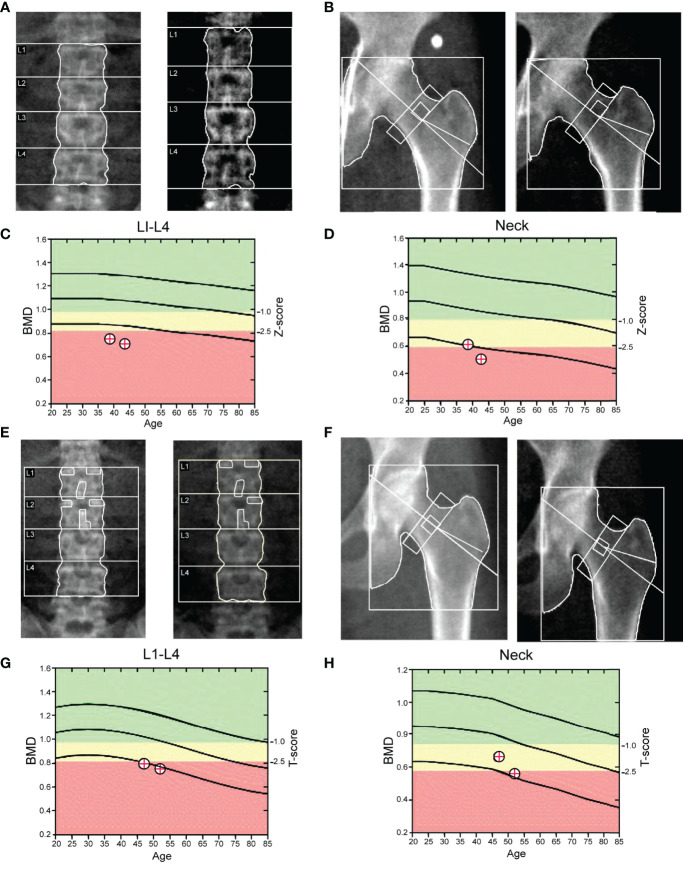
DXA scans of the lumbar spine and femoral neck. **(A–D)** Homozygous man (38 years); the Z-scores for lumbar spine (L1–L4) and femoral neck DXA were -3.0 and −1.9, respectively. The diagnosis by DXA was osteopenia. During the follow-up, the Z-scores for the lumbar spine (L1–L4) and femoral neck DXA were -3.3 and −2.2, respectively. **(E–H)** Homozygous woman (47 years). The T-scores for the lumbar spine (L1–L4) and femoral neck DXA were -2.6 and -1.7, respectively. The diagnosis by DXA was osteopenia. During the follow-up, the T-scores for the lumbar spine (L1–L4) and femoral neck DXA were -3.1 and −2.4, respectively.

In the present study, 10 individuals (four males and six females) with the homozygous p.Ser267Phe mutation in NTCP were recruited. All homozygous individuals had a marked elevation in the levels of BAs and low levels of vitamin D in serum. In the 5-year follow-up, these patients did not exhibit clinical signs of cholestatic jaundice, pruritis, fat malabsorption, liver dysfunction, or nervous system abnormalities. Their abdominal ultrasonography showed a liver with normal size and parenchyma without any biliary tract abnormalities. Then, the ultrasound examinations showed clear gallbladder polyps in two males and one female. One male also presented a gallbladder with multiple gallstones. Detailed clinical features are presented in **Table 1**. Since these homozygous patients had vitamin D deficiency, we further assessed the bone mineral density (BMD) of seven volunteers using dual-energy X-ray absorptiometry (DXA). The BMD results indicated that one adult man and one postmenopausal woman had osteoporosis, one adult man had a bone density decline, and four adult women without menopause had bone density within the normal range. Homozygous individuals with osteoporosis did not regularly use vitamin D and calcium tablet supplements and did not receive anti-osteoporosis treatment. Five years later, the follow-up DXA scan showed a dramatic BMD decline in two homozygous individuals (**Figures 1A-H**). However, the other homozygous individuals did not agree to repeat the BMD test. None of the homozygous individuals presented with multiple bone pains, height loss, or fractures. During the 5-year follow-up, we found that the most common phenotypic features of NTCP deficiency in adults were hypercholanemia, vitamin D deficiency, bone loss, and gallbladder abnormalities.

**Table 1:** Clinical characteristics of 10 homozygous patients.

Routine Clinical chemistry	Unit	Mean	Range (min to max)	Reference
**Liver standard tests**				
ALT	U/L	21.50	9.00-29.00	3.00-35.00
AST	U/L	24.00 19.41	16.00-**44.00 **(Male); 15.00-27.00 (Female)	15.00-40.00 (Male); 13.00-35.00 (Female)
TBIL	μmol/L	9.12	4.60-16.5	4.00-23.90
DBIL	μmol/L	2.64	1.20-5.40	0.00-6.80
ALB	g/L	45.00	39.00-50.40	36.00-51.00
GGT	U/L	22.30	2.00-55.00 (Male); 8.00-45.00 (Female)	10.00-60.00 (Male) 7.00-45.00 (Female)
ALP	U/L	57.90	37.00-103.00 (Male); 37.00-79.60 (Female)	45.00-125.00 (Male) 35.00-135.00 (Female)
TBA	μmol/L	**40.54**	**19.50-138.3**	0-14.00
**Vitamins levels**				
25(OH)Vitamin D3	ng/ml	**20.29**	**7.39**-39.08	25-50
Vitamin A	ng/ml	488.56	**290.15**-1005.68	325-780
Vitamin E	ng/ml	7.79	**4.30**-12.99	5.5-17
Vitamin K1	ng/ml	0.85	0.30-1.18	0.1-2.2
Vitamin B2	ng/ml	7.22	3.62-15.76	1-19
Vitamin B5	ng/ml	64.01	39.30-104.14	37-147
**Blood lipids**				
Total Cholesterol	mmol/L	4.73	2.27-**6.65**	3.10-5.70
Triglyceride	mmol/L	1.19	0.44-**3.62**	0.34-1.92
HDLC	mmol/L	1.31	0.58-**2.06**	0.78-2.00
LDLC	mmol/L	2.30	1.23-**4.59**	2.07-3.10

ALT, Alanine transaminase; AST, Aspartate transaminase; TBIL, Total bilirubin; DBIL, direct bilirubin; ALB, Albumin; GGT, gamma-glutamyl transferase; ALP, alkaline phosphatase; TBA, Total bile acid; HDLC, high-density lipoprotein cholesterol; LDLC, low-density lipoprotein cholesterol. Data that exceeds the reference range is shown in bold.

### The Profile of Serum BAs Was Significantly Altered in Homozygotes Carrying the NTCP p.Ser267Phe Mutation

**Figure 2:**
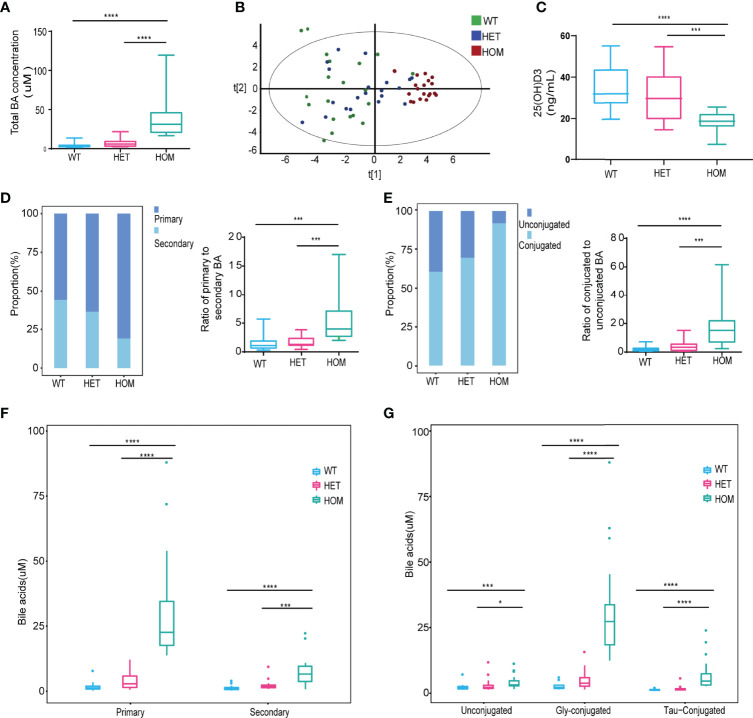
The profile of bile acids (BAs) in the serum was significantly altered in homozygotes carrying the p.Ser267Phe mutation. **(A)** Levels of total BAs in serum. **(B)** PCA of WT, HET, and HOM individuals using all BAs identified. **(C)** Levels of 25(OH)D3 in serum. **(D)** Stack bar plot representing the proportion of total primary and secondary BAs, and box and whisker plots for the primary to secondary BAs ratio. **(E)** Stack bar plot representing the proportion of total conjugated and unconjugated BAs, and box and whisker plots for the conjugated to unconjugated BAs ratio. **(F)** Primary and secondary BAs. **(G)** Concentrations of unconjugated, Gly-conjugated, and Tau-conjugated BAs in serum. *, p < 0.05; ***, p < 0.001; and ****, p < 0.0001.

Next, to characterize the profile of BAs associated with the NTCP p.Ser267Phe variant, we analyzed the serum BAs from 10 HOM, 20 HET, and 20 WT individuals. The demographic and clinical characteristics of study participants are described in **Table S2**. The total concentration of BAs significantly increased in the serum of the HOM group compared to the WT or HET groups (**Figure 2A**). We observed a clear separation among the three groups using a Principal Component Analysis (PCA) model established with the BAs identified [**Figure 2B**; t (1) = 0.386, t (2) = 0.195]. The profile of 41 BAs identified are listed in **Table S3**. Then, we compared the vitamin D levels. We found that the levels of 25(OH)D3 (the active form of vitamin D) were significantly lower in the HOM group compared to the WT or HET groups (**Figure 2C**). Additionally, the proportion of primary BAs increased from WT to HET to HOM patients. In the HOM group, the ratio of primary to secondary BAs was also significantly increased compared to the WT or HET groups (**Figure 2D**). The proportion of conjugated BAs in serum increased from WT to HET to HOM patients. Compared to the WT or HET groups, the ratio of conjugated to unconjugated BAs was also higher in the HOM group (**Figure 2E**). Moreover, the concentrations of main conjugated BAs, including GCA, TCA, GHCA, THCA, TDCA, GCDCA, GDCA, and TCDCA, significantly increased in the HOM group compared to the WT or HET group (**Table S3**). Similarly, the levels of primary, secondary, glycine (Gly)- and taurine (Tau)-conjugated BAs were significantly elevated in the HOM group compared to the WT and HET groups (**Figures 2F, G**). Finally, the levels of unconjugated BAs were slightly elevated in the HOM group compared to the WT and HET groups (**Figure 2G**). Overall, the profile of BAs in the serum of homozygotes carrying the NTCP p.Ser267Phe mutation was significantly altered and marked by both elevated proportion and concentration of primary and conjugated BAs.

### NTCP Deficiency Aggravates Osteoporosis in OVX Mice

**Figure 3:**
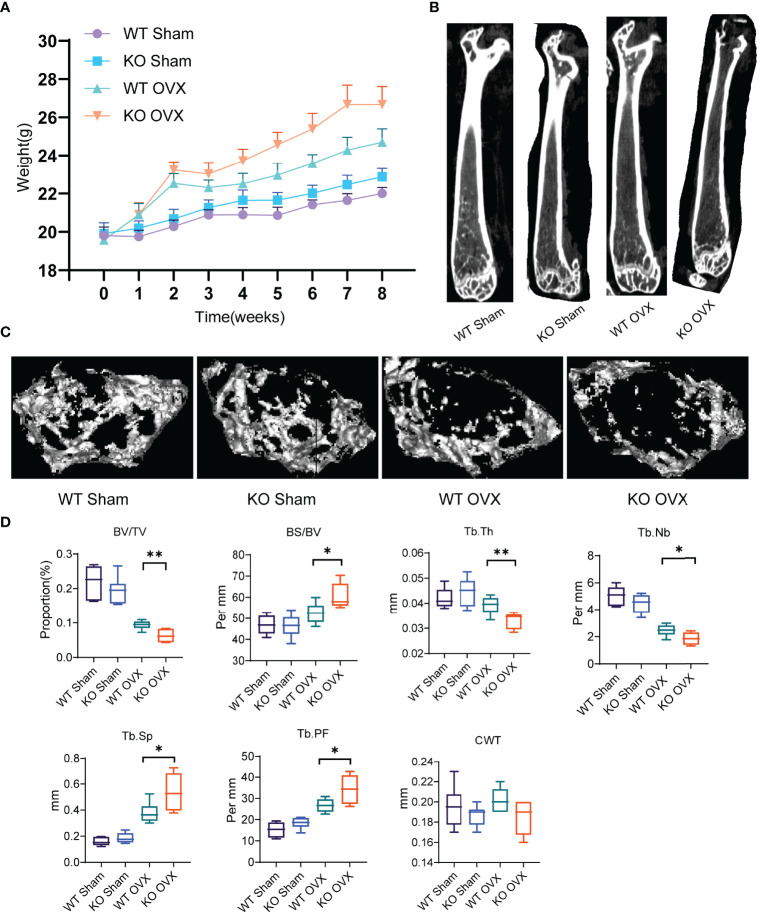
NTCP deficiency aggravated osteoporosis in OVX mice. **(A)** Bodyweight of WT SHAM, KO SHAM, WT OVX, and KO OVX mice over 8 weeks after the establishment of OVX or SHAM models. **(B, C)** Representative micro-CT images showing vertical **(B)** and cross **(C)** sections of the femur of WT SHAM, KO SHAM, WT OVX, and KO OVX mice. **(D)** NTCP deficiency aggravated osteoporosis in OVX mice. BV/TV, bone volume/tissue volume; BS/BV, bone surface area/bone volume; Tb.Th, trabecular thickness; Tb.Nb, trabecular number; Tb.Sp, trabecular spacing; Tb.PF, trabecular pattern factor; CWT, cortical wall thickness. n = 6 mice per group. *, p < 0.05 and **, p < 0.01.

Furthermore, to understand the phenotypic characteristics of NTCP deficiency, we generated and characterized NTCP deficient mice. The establishment of NTCP global knockout (KO) mice using CRISPR/Cas9 nickase technology is described in **Figure S1**. To assess the relationship between NTCP deficiency and osteoporosis, we used a classic approach to induce postmenopausal osteoporosis by constructing an ovariectomy (OVX)-induced osteopenic model (18). The WT and NTCP-KO female mice were operated (sham or OVX) at 8 weeks. After 8 weeks, mice were euthanized and all examinations were performed. Compared to the sham group, the OVX group presented a significant weight gain (**Figure 3A**). Next, we measured the trabecular and cortical bone mass of the femurs of WT and NTCP-KO mice using micro-CT. Vertical sections of the whole femur and three-dimensional images are shown in **Figures 3B, C**. We found that the bone volume/tissue volume (BV/TV) ratio, trabecular thickness (Tb.Th), and trabecular number (Tb.Nb) were significantly lower in NTCP-KO OVX mice than in WT OVX mice (**Figure 3D**). Additionally, we observed a higher bone surface area/bone volume (BS/BV) ratio, trabecular spacing (Tb.Sp), and trabecular pattern factor (Tb.PF) in NTCP-KO OVX mice compared with WT OVX mice. However, the trabecular thickness (Tb.Th) did not differ between WT and NTCP-KO OVX mice. The morphological parameters from micro-CT analyses of WT mice did not significantly differ from NTCP-KO sham mice (**Figure 3D**).

**Figure 4:**
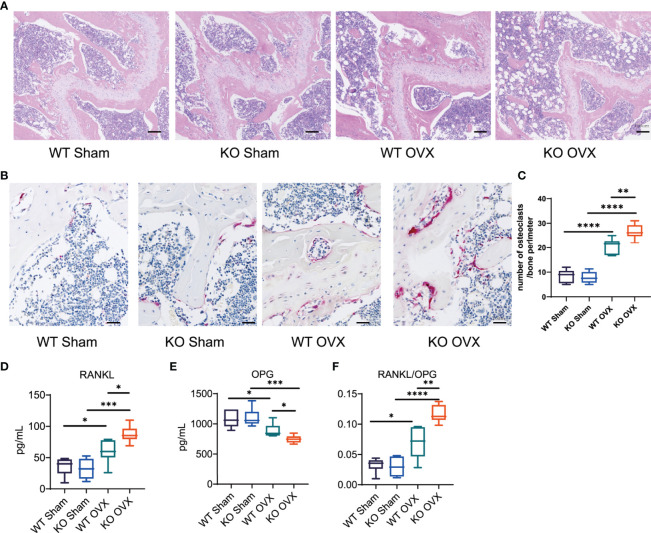
Impacts of NTCP deficiency on osteogenesis and osteoclastogenesis in OVX mice. **(A–C)** Representative H&E-stained **(A)** and TRAP-stained **(B)** femur tissue sections from WT SHAM, KO SHAM, WT OVX, and KO OVX mice. The ratio of TRAP-positive cells in the field was counted in each group **(C)**. **(D–F)** Levels of RANKL and OPG, and RANKL to OPG ratio of WT SHAM, KO SHAM, WT OVX, and KO OVX mice. n = 6 mice per group. *, p < 0.05; **, p < 0.01; ***, p < 0.001; and ****, p < 0.0001.

Next, we tested the impacts of NTCP deficiency on osteogenesis and osteoclastogenesis in OVX mice. Regarding the osteoporosis pathological phenotypes in femur tissue sections, the H&E staining and histomorphology analyses revealed decreased bone mass with loss of trabecular bone underneath growth plates as well as increased marrow adipocytes with enhanced adipogenic potential, and decreased osteogenic differentiation potential in NTCP-KO OVX mice compared with WT OVX mice (**Figure 4A**). We also analyzed the osteoclasts in the bones of OVX mice by TRAP staining. Compared to OVX WT mice, NTCP-KO mice presented a higher number of TRAP+ osteoclasts, indicating that they had a faster bone tissue breakdown (**Figures 4B, C**). Further, we analyzed the OPG-receptor activator of the RANKL system since this axis plays a crucial role in bone remodeling. The circulating levels of RANKL in NTCP-KO mice were significantly higher than in WT mice after OVX (**Figure 4D**), while the OPG showed an opposite trend (**Figure 4E**). Finally, the OPG/RANKL ratio showed more clear results (**Figure 4F**). Altogether, these results demonstrated that NTCP deficiency aggravated the osteoporotic phenotype induced by estrogen withdrawal in mice by reducing osteoclast formation and enhancing osteoblast formation.

### NTCP Deficiency Increased the Levels of BAs and Decreased the Levels of Vitamin D in the Serum of Mice

**Figure 5:**
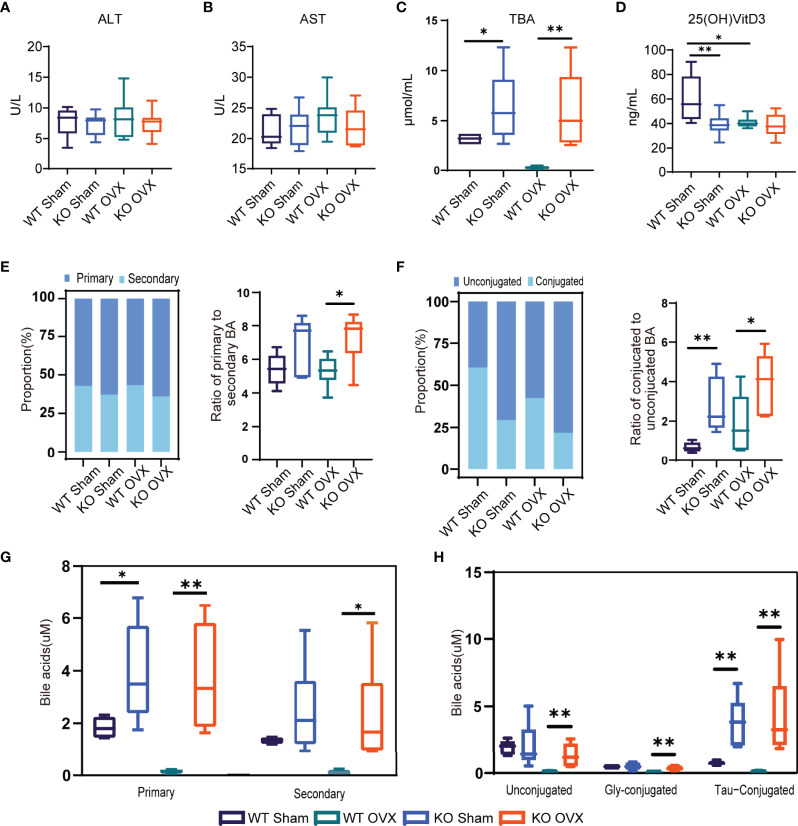
NTCP deficiency increased the levels of bile acids (BAs) in the serum of mice. **(A–D)** Levels of ALT, AST, TBA, and 25(OH)D3 in serum of WT SHAM, KO SHAM, WT OVX, and KO OVX mice. n = 6 mice per group. **(E)** Stack bar plot representing the proportion of total primary and secondary BAs and box and whisker plots for the primary to secondary BAs ratio. **(F)** Stack bar plot representing the proportion of total conjugated and unconjugated BAs and box and whisker plots for the conjugated to unconjugated BAs ratio. **(G)** Primary and secondary BAs. **(H)** Concentrations of unconjugated, Gly-conjugated, and Tau-conjugated BAs in serum. *, p < 0.05 and **, p < 0.01.

Compared to WT mice, the NTCP-deficient mice had similar levels of ALT and AST liver function (**Figures 5A, B**), elevated levels of serum BAs (**Figure 5C**), and reduced levels of 25(OH)VitD3 (**Figure 5D**). Then, we characterized the levels of circulating BAs in NTCP-KO mice. The NTCP-KO OVX mice presented a significantly higher ratio of primary to secondary BAs compared to WT OVX mice (**Figure 5E**). The proportion of conjugated BAs in the serum increased from WT to NTCP-KO mice, similar to the ratio of conjugated to unconjugated BAs in both OVX and sham models (**Figure 5F**). Finally, the levels of primary, secondary, unconjugated, and conjugated BAs were higher in NTCP-KO OVX mice compared to WT OVX mice (**Figures 5G, H**). Overall, NTCP deficiency increased the levels of BAs in the serum and decreased the levels of vitamin D in mice, consistent with the previous results for NTCP p.Ser267Phe homozygous individuals.

## Discussion

In the current study, we conducted a comprehensive medical evaluation of 10 adults with NTCP deficiency. During the 5-year follow-up, we found that the most common phenotypic features of NTCP deficiency in adults were hypercholanemia, vitamin D deficiency, bone loss, and gallbladder abnormalities. We also demonstrated that the loss of NTCP function aggravated osteoporosis induced by estrogen deficiency in an OVX-induced osteopenic model.

According to previous reports, seven different mutations of the *SLC10A1* gene have been linked to NTCP deficiency. The single-nucleotide polymorphism (SNP) rs2296651, NTCP p.Ser267Phe mutation, is common in Southeast Asia with a minor allele frequency (MAF) of 8%-12% (8, 15), thereby affecting a considerable number of NTCP-deficient individuals in the population. Here, we described the clinical features of NTCP p.Ser267Phe homozygous adults. These individuals presented with hypercholanemia, vitamin D deficiency, bone loss, and gallbladder abnormalities. During the follow-up, the hypercholanemia was asymptomatic and well-tolerated. However, our results indicated that hypercholanemia is accompanied by vitamin D deficiency and osteopenia or osteoporosis. Although individuals with NTCP deficiency had osteoporosis or decreased bone mass during the follow-up, they did not receive standard anti-osteoporosis treatment due to their asymptomatic nature. Moreover, Deng et al. reported that NTCP-deficient pediatric patients with cholestasis are more prone to vitamin D deficiency during early infancy (19). Previous studies have also described that infants with NTCP deficiency presented indirect hyperbilirubinemia, transient cholestasis, or growth retardation, besides exhibiting elevated levels of BAs (7, 20–22). Additionally, the elevation of total BAs in pediatric patients is much higher than in adults with NTCP deficiency. The possible explanation for the more significant effects of NTCP deficiency on infants might be that alternative BA transporters, such as organic anion transporting polypeptides (OATPs), would not compensate for the NTCP deficiency at the beginning. Also, different from indirect hyperbilirubinemia and transient cholestatic jaundice in some pediatric patients, we did not observe elevated bilirubin in adult homozygotes. Additionally, 30% (3/10) of the homozygous individuals presented gallbladder abnormalities, including gallbladder polyps and gallstones. However, the gallbladder polyps did not increase during follow-up. Previous studies have also suggested that NTCP deficiency can lead to gallbladder abnormalities in both mice and humans (23, 24). Therefore, we recommend regular abdominal ultrasounds to detect changes in the gallbladder of NTCP-deficient patients.

Consistent with previous reports (8, 14, 15, 19), we observed hypercholanemia in patients with NTCP deficiency caused by the p.Ser267Phe mutation. First, the NTCP deficiency led to an increase in total circulating BAs, marked by elevated concentration of primary and conjugated BAs. The fractionated Gly- and Tau-conjugated BAs were also elevated in p.Ser267Phe mutation homozygotes. NTCP mainly functions as a sodium-dependent transporter of conjugated BAs. The NTCP p.Ser267Phe mutation has been described to cause almost complete loss of this transport activity (11–13). The profile of BAs in NTCP-deficient individuals demonstrated the primary role of NTCP in hepatic BA clearance and reabsorption of conjugated BAs. NTCP is considered the primary transporter and OATPs are an alternative for hepatic uptake of BAs (1, 5). Since unconjugated BAs are poor substrates for NTCP (25), increased levels of unconjugated BAs in the serum might indicate a competitive inhibition of OATPs by conjugated BAs. These results also indicate a limited role of alternative hepatic BA uptake mechanisms to maintain the normal cycling of enterohepatic BA in humans. Whether the synthesis of BAs is affected by NTCP mutations requires further investigation. In 2017, Davor et al. reported that enterocytes sense elevated levels of conjugated BAs in the systemic circulation to induce FGF15/19, which reduces hepatic BA synthesis and modulates BA transporters (1). A previous study reported that the fecal excretion of BAs decreased and the urinary concentration of BAs increased in NTCP-KO mice with hypercholanemia (6). These results are consistent with another study that showed increased urine bile salt excretion in NTCP-deficient patients (7). Whether the fecal and renal excretion of BAs in NTCP p.Ser267Phe homozygotes are similar to NTCP-KO mice requires further study. Various inherited and acquired conditions affecting BA homeostasis can cause hypercholanemia (8, 26). BAs possess detergent-like properties and are thought to be cytotoxic. For example, previous studies have reported that excessive concentrations of BAs can induce hepatocyte injury by activating the death receptor pathway. Moreover, elevated concentrations of BAs are associated with cholestatic liver diseases, liver cirrhosis, hepatocellular carcinoma, nonalcoholic fatty liver disease, and hepatic encephalopathy (27–30). Although the homozygotes in this study presented elevated levels of circulating BAs, we did not detect liver damage. Interestingly, the inhibition of NTCP can have hepatoprotective effects by reducing BA load in hepatocytes and increasing the biliary phospholipid/BA ratio (31). Previously, NTCP-KO mice presented reduced diet-induced obesity and hepatic steatosis by simultaneously dampening intestinal fat absorption and increasing energy expenditure (32). A recent study has also indicated that the possible mechanism for the tolerance to hypercholanemia of NTCP p.Ser267Phe homozygotes might be the increased sulfation of BAs to detoxify and eliminate them (33). These results indicate the possibility of a reduced intrahepatic cytotoxic accumulation of BAs and hepatic inflammation or hepatocyte injury.

Furthermore, BAs are essential for the absorption of lipids and lipid-soluble nutrients from the intestine (34, 35). In the present study, we found that homozygous patients presented vitamin D deficiency but the levels of other fat-soluble vitamins (A, E, and K) did not decrease. Previous studies have also reported that NTCP deficiency is associated with vitamin D deficiency (15, 19). This might be caused by the impaired enterohepatic circulation of BAs due to loss of NTCP function. Hence, decreased BA levels in the intestines of mice might cause insufficient absorption of vitamin D in the intestines, which in turn leads to vitamin D deficiency. The vitamin D obtained from diet and skin is hydroxylated into 25-hydroxyvitamin D [25(OH)D] in the liver. Next, 25(OH)D is converted into its active form, 1,25(OH)D, by a second hydroxylation in the kidney (36). Vitamin D stimulates the vitamin D receptor (VDR) in osteoblasts, promotes RANKL-mediated osteoclastogenesis, and stimulates the production of the receptor activator of NF-κβ (RANK), and OPG (37). Therefore, liver diseases negatively affect the production of active vitamin D metabolites and result in abnormal bone metabolism. Hepatic osteodystrophy, including osteopenia, osteoporosis, and osteomalacia, is a common complication in chronic liver disease patients and is related to the duration and severity of cholestasis, vitamin D deficiency, alcohol consumption, and calcium malabsorption (38, 39). Low bone formation is the main pathophysiological mechanism behind osteoporosis and is associated with a transient increase in bone resorption (38). In the present study, we found that the NTCP deficiency was associated with bone loss. Thus, homozygous individuals are more prone to bone loss and osteoporosis. Finally, we recommend monitoring the levels of BAs and vitamin D and the bone density in NTCP-deficient individuals, and performing medical intervention if necessary. Since the inhibition of NTCP is a pharmacological target in the treatment of hepatitis B and D viruses infections, hepatic fibrosis, and hepatosteatosis (40), monitoring the levels of total BAs and vitamin D and the bone density should also be considered during NTCP inhibitor development.

Our current study also has some limitations. First, we did not analyze the liver pathologies of homozygotes. However, all homozygotes were asymptomatic with a relatively normal liver function and were not indicated for liver biopsy. Hence, more assessments of the liver status using non-invasive methods are needed for a long-term follow-up. Second, we found that the NTCP p.Ser267Phe mutation was related to bone loss, but we did not analyze its relevant molecular mechanisms, especially how this mutation affects bone metabolism. We could not demonstrate a direct relation between NTCP deficiency and increased osteoclastogenesis and diminished bone mass *in vivo*. Since we used a global KO mice model, we could not fully exclude the effects of other cell types expressing NTCP on bone metabolism and future studies are required to corroborate our findings. Finally, we only tested the profile of BAs in the serum and did not analyze it in feces and urine, which are important excretion routes of BAs in the metabolism. Therefore, further studies are needed to explore the BA excretion of BAs in NTCP-deficient individuals.

In summary, we expanded the knowledge regarding NTCP mutations and provided comprehensive insights into the long-term clinical manifestations and prognosis of NTCP-deficient individuals. We recommend monitoring the levels of BAs and vitamin D, bone density, and abdominal ultrasounds of NTCP-deficient individuals. If necessary, medical interventions should be performed.

## Data Availability Statement

The original contributions presented in the study are included in the article/**Supplementary Materials**, further inquiries can be directed to the corresponding author/s.

## Ethics Statement

The studies involving human participants were reviewed and approved by The Third Affiliated Hospital of Sun Yat-sen University [(2015) 2-155]. The patients/participants provided their written informed consent to participate in this study. The animal study was reviewed and approved by Institutional Animal Care and Use Committee from South China Agricultural University[2021D078].

## Author Contributions

FY, WX, LiW, YC, and LP planned the study; FY, LiW, SZ, LuW, LY, and LP recruited the cohort and followed up; WX, LY, and LuW collected the clinical data; FY, LiW, WW, and YZ performed the mouse experiment; LY, SZ, and FY performed statistical analyses; FY, WX, and LiW wrote the first draft of the manuscript, tables and figures; YC and LP dealt with the final typing and is responsible for the overall content of the manuscript acting as guarantor. All authors have contributed significantly to the manuscript.

## Funding

This study was supported by grants from the National major science and technology project for the prevention and treatment of AIDS and viral hepatitis (2018ZX10302204-002 to LP), Natural Science Foundation of China (No. 81873572 and 82070611 to LP), Science and Technology Planning Project of Guangdong Province, China (2019B020228001 to YC), Guangzhou Science and Technology Plan Projects (No. 202102080064 to WX, 202102010204 to LP, 201804010474 to YC), Sun Yat-Sen University Clinical Research 5010 Program (2020007 to LP, 2016009 to YC), the Five-Year Plan of Third Affiliated Hospital of Sun Yat-Sen University (K00006 to LP) and China Postdoctoral Science Foundation (2020T130149ZX and 2020M672975 to FY).

## Conflict of Interest

The authors declare that the research was conducted in the absence of any commercial or financial relationships that could be construed as a potential conflict of interest.

## Publisher’s Note

All claims expressed in this article are solely those of the authors and do not necessarily represent those of their affiliated organizations, or those of the publisher, the editors and the reviewers. Any product that may be evaluated in this article, or claim that may be made by its manufacturer, is not guaranteed or endorsed by the publisher.
