# Supplementary material for: NTCP Deficiency Affects the Levels of Circulating Bile Acids and Induces Osteoporosis
Source: Front Endocrinol (Lausanne). 2022 Jul 22;13:898750. doi: 10.3389/fendo.2022.898750 (PMC9353038; doi:10.3389/fendo.2022.898750)
Supplement: Supplementary file 1 [file DataSheet_1.docx]

Supplementary Material

##
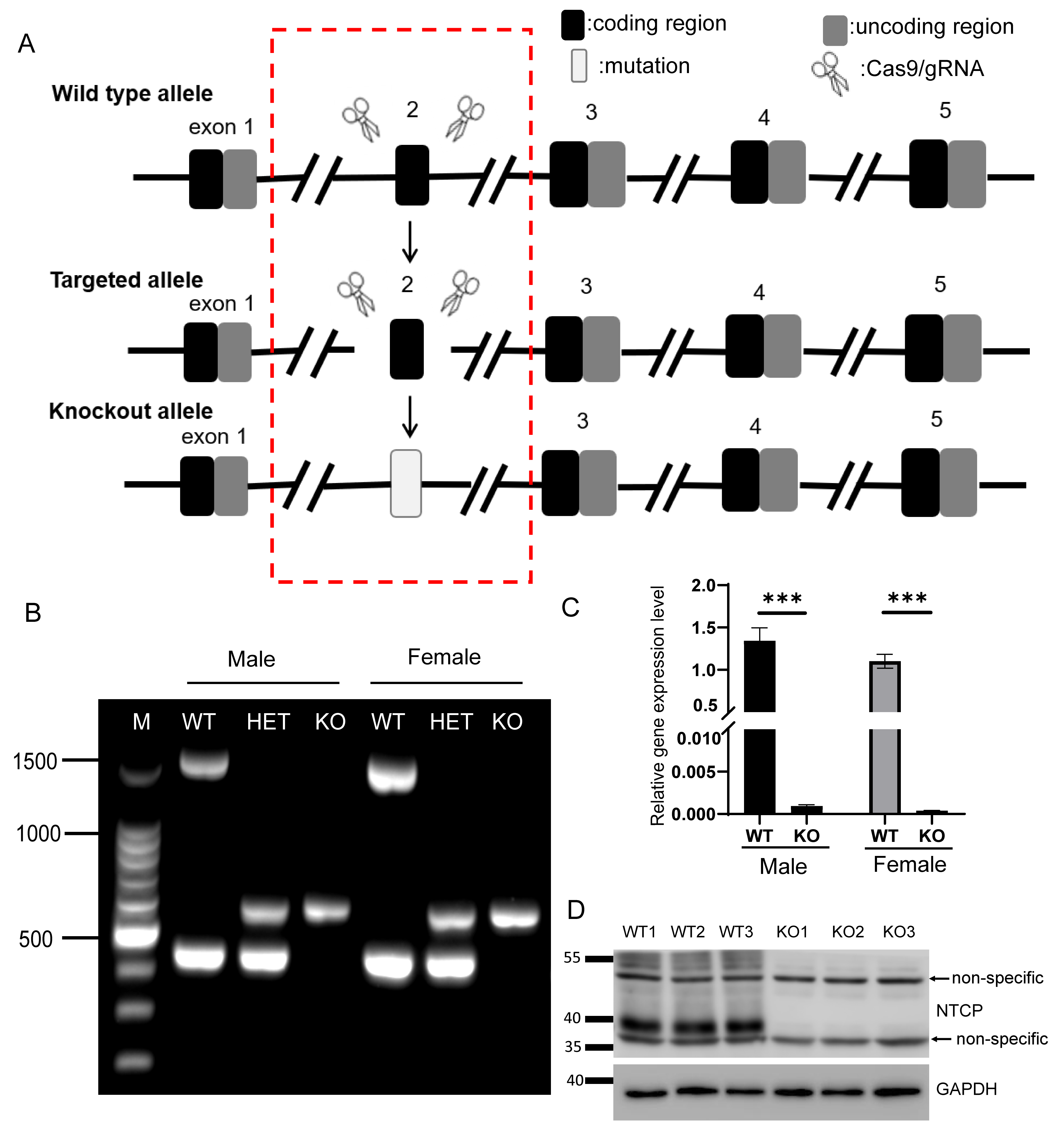
Supplementary Figures

**Supplementary Figure 1.** **Generation of NTCP-KO mice mediated by CRISPR/Cas9.**

(A) Schematic illustration of the generation of NTCP-KO mice. Two sgRNAs were designed to delete exons 1 to 3 of the SLC10A1 gene. (B) Genomic DNA was extracted for genotyping. Homozygous WT (+/+) mice, heterozygous (+/-), or homozygous KO (-/-) male and female mice are shown. The SLC10A1 WT (1866 and 435-bp) and targeted (854-bp) alleles were detected by PCR. (C) The mRNA expression of SLC10A1 in the livers of male and female WT and SLC10A1-KO mice was analyzed by qPCR. SLC10A1 expression values were calculated relative to ACTB and normalized to WT mice. Data are presented as means ± SDs (six mice in each group). (D) NTCP protein levels (35-40 kDa) in the liver of WT and KO male mice (n = 3) by Western blot. Total protein was treated with PNGase F (New England Biolabs, Ipswich, MA). Antibodies against the NTCP (PB9745, Boster Biotechnology, Wuhan, China) were used at the dilution of 1:1,000.

**1.2 Supplementary tables**

**Table S1 List of 41 bile acid (BA) standards and the abbreviation**

| NO. | BAs | Abbr |
| --- | --- | --- |
| 1 | β-ursocholic acid | βUCA |
| 2 | ursocholic acid | UCA |
| 3 | glycoursodeoxycholic acid | GUDCA |
| 4 | glycohyodeoxycholic acid | GHDCA |
| 5 | glycohyocholic acid | GHCA |
| 6 | glycocholic acid | GCA |
| 7 | tauroursodeoxycholic acid | TUDCA |
| 8 | tauro α-muricholic acid | TαMCA |
| 9 | taurohyocholic acid | THCA |
| 10 | taurocholic acid | TCA |
| 11 | dehydrolithocholic acid | dehydroLCA |
| 12 | allolithocholic acid | alloLCA |
| 13 | isolithocholic acid | isoLCA |
| 14 | lithocholic acid | LCA |
| 15 | 23-nordeoxycholic acid | NorDCA |
| 16 | 7-ketolithocholic acid | 7-ketoLCA |
| 17 | 12-ketolithocholic acid | 12-ketoLCA |
| 18 | chenodeoxycholic acid | CDCA |
| 19 | deoxycholic acid | DCA |
| 20 | glycolithocholic acid | GLCA |
| 21 | lithocholic acid-3-sulfate | LCA-3S |
| 22 | 6-ketolithocholic acid | 6-ketoLCA |
| 23 | 3β-ursodeoxycholic acid | βUDCA |
| 24 | 3β-chenodeoxycholic acid | βCDCA |
| 25 | 3β-deoxycholic acid | βDCA |
| 26 | ursodeoxycholic acid | UDCA |
| 27 | α-hyodeoxycholic acid | HDCA |
| 28 | norcholic acid | NorCA |
| 29 | 7-ketodeoxycholic acid | 7-DHCA |
| 30 | 3-dehydrocholic acid | 3-DHCA |
| 31 | 3β-cholic acid | βCA |
| 32 | β-muricholic acid | βMCA |
| 33 | hyocholic acid | HCA |
| 34 | allocholic acid | ACA |
| 35 | cholic acid | CA |
| 36 | glycochenodeoxycholic acid | GCDCA |
| 37 | glycodeoxycholic acid | GDCA |
| 38 | taurochenodeoxycholic acid | TCDCA |
| 39 | taurodeoxycholic acid | TDCA |
| 40 | glycolithocholic acid-3-sulfate | GLCA-3S |
| 41 | chenodeoxycholic acid-3-β-d-glucuronide | CDCA-3Gln |

**Table S2 Demographic and clinical characteristics of study participants**

|  | WT group | HET group | HOM group |
| --- | --- | --- | --- |
| Number | n=20 | n=20 | n=10 |
| Sex(M/F) | (8/12) | (8/12) | (4/6) |
| Age(years) | 37.88±12.8 | 32.78±12.74 | 39.3±14.94 |
| BMI(kg/m^2^） | 21.51±2.78 | 21.31±2.05 | 21.46±2.53 |
| ALT(U/L) | 38.18±52.35 | 19.83±4.16 | 21.5±6.81 |
| AST(U/L) | 48.65±116.76 | 12.78±7.13 | 16.15±5.81 |
| TBIL(umol/L) | 17.98±22.81 | 12.36±4.77 | 9.12±3.18 |
| DBIL(umol/L) | 11.63±18.85 | 4.34±1.89 | 2.64±1.21 |
| ALB(g/L) | 44.99±3.06 | 45.54±3.37 | 45.83±3.2 |
| GGT(U/L) | 40.75±43.95 | 19.71±8.98 | 22.3±13.56 |
| ALP(U/L) | 69.3±37.02 | 60.18±21.63 | 57.9±21.12 |
| CHE(U/L) | 7348.25±1958.72 | 8256.67±1365.4 | 8512±1591.35 |
| BUN(mmol/L) | 4.82±1.23 | 4.19±1.34 | 4.33±0.96 |
| CREA(umol/L) | 69.8±19.94 | 69.8±6.91 | 70.27±19.95 |

Note: Data are represented as Mean ± SD. ALT, Alanine transaminase; AST, Aspartate transaminase; TBIL, Total bilirubin; DBIL, direct bilirubin; ALB, Albumin; ALP, alkaline phosphatase; GGT, gamma-glutamyl transferase; TBA, Total bile acid; CHE, cholinesterase; BUN, Blood urea nitrogen; CREA, creatinine.

**Table S3 BA profiles among NTCP WT, HET and HOM groups**

| BAs | WT group(n=20) | HET group(n=20) | HOM group(n=10) | *P*-value^§^ | *P*-value**^‡^** |
| --- | --- | --- | --- | --- | --- |
| βUCA | 0.43 (0.04,2.56) | 0.23(0.04,0.87) | 0.12(0.04,0.46) | 0.379 | 0.756 |
| UCA | 0.04 (0.01, 1.57) | 0.04(0.00,1.10) | 0.02(0.00,0.79) | 0.379 | 0.579 |
| **GUDCA** | 129.1(69.43,333.5) | 264.00(124.10,491.50) | 343.30 (158.50,552.90) | 0.005 | 0.229 |
| **GHDCA** | 3.81(2.62,5.47) | 3.90(3.21,5.80) | 6.92 (4.77,12.77) | 0.006 | 0.033 |
| **GHCA** | 18.94 (12.11,28.36) | 28.88(16.36,51.71) | 157.50(103.40,250.90) | <0.001 | <0.001 |
| **GCA** | 99.81(72.0,198.0) | 336.80(275.20,747.70) | 7701.00 (5391.0,13420.0) | <0.001 | <0.001 |
| **TUDCA** | 2.07 (1.02,8.36) | 8.07(5.49, 16.91) | 11.88(7.91,37.34) | 0.001 | 0.156 |
| **TαMCA** | 7.03 (4.05,10.87 ) | 11.05(8.05, 24.19) | 29.26(5.25,126.90) | 0.010 | 0.365 |
| **THCA** | 3.43 (1.80 ,5.58) | 6.11(3.07,10.14) | 30.64(14.92,72.28) | <0.001 | <0.001 |
| **TCA** | 12.87 (6.13,34.40) | 49.67(22.70, 89.20) | 1663.00(893.80,3360.00) | <0.001 | <0.001 |
| dehydroLCA | 3.42 (2.20,3.85) | 3.04(2.44,3.66) | 2.93(2.31,4.81) | 0.745 | 0.818 |
| alloLCA | 2.08 (1.04, 3.47) | 1.31(0.98, 2.42) | 1.53(1.06,3.10) | 0.552 | 0.626 |
| isoLCA | 17.84 (2.27, 40.29) | 11.93(4.45, 18.08) | 14.26(2.36,39.40) | 0.989 | 0.715 |
| LCA | 10.86 (1.76, 18.17) | 10.55(3.76,13.09) | 7.19(2.01,22.77) | 0.756 | 0.925 |
| NorDCA | 4.18 (1.66, 6.07) | 3.20(1.64 ,3.86) | 2.96(1.43,3.50) | 0.156 | 0.337 |
| 7-ketoLCA | 12.73 (8.40, 22.01) | 11.89(7.20 ,22.39) | 13.18 (7.68, 29.64) | 0.735 | 0.655 |
| 12-ketoLCA | 7.82(2.47, 17.09) | 8.79(5.88, 17.38) | 7.58(2.21, 18.80) | 0.860 | 0.543 |
| CDCA | 286.50(189.20,545.5) | 338.70(183.6, 814.7) | 672.80(354.4,1250.0) | 0.026 | 0.102 |
| DCA | 278.9 (83.02, 438.1) | 310.50(191.60, 699.60) | 444.70(85.01, 966.10) | 0.273 | 1.000 |
| GLCA | 5.18(1.18, 10.72) | 11.38(4.55, 15.57) | 8.75 (1.58, 17.35) | 0.239 | 0.715 |
| LCA_S | 3.95(1.25, 9.90) | 4.20(1.95, 10.86) | 4.13 (1.77,10.83) | 0.655 | 0.989 |
| LCA-3S | 3.70(2.21, 4.75) | 4.16(2.70, 5.36) | 3.43(2.79, 4.69) | 0.871 | 0.508 |
| 6-ketoLCA | 123.50(73.14,632.1) | 212.60(134.80,417.40) | 167.60 (98.09,301.50) | 0.914 | 0.298 |
| βUDCA | 83.17(54.21, 203.80) | 108.70(51.24,217.90) | 141.50(69.75,210.90) | 0.409 | 0.617 |
| βCDCA | 32.75(14.32, 76.99) | 42.28(16.62, 94.92) | 60.88(5.67, 99.30) | 0.978 | 0.617 |
| βDCA | 92.68(29.57, 216.00) | 92.13(70.92, 164.50) | 77.73 (44.98, 246.20) | 0.617 | 0.675 |
| HDCA | 5.12(2.63, 11.41) | 5.80(3.96, 11.48) | 6.14(4.14, 11.52) | 0.365 | 0.715 |
| NorCA | 3.41(2.44, 4.81) | 5.72(3.25, 7.40 ) | 4.53(3.45,5.90) | 0.152 | 0.525 |
| 7-DHCA | 2.96(1.69, 5.34) | 3.85(1.59, 8.25) | 5.41(1.79, 28.93) | 0.091 | 0.208 |
| **3-DHCA** | 3.27(1.79, 4.31) | 5.32(3.48, 9.19) | 25.44 (14.40, 47.21) | <0.001 | <0.001 |
| βCA | 6.05(5.17, 8.56) | 6.78(5.50, 10.80) | 6.12(4.94, 9.52) | 0.850 | 0.379 |
| βMCA | 0.99(0.44, 5.27) | 2.22(0.32, 7.32) | 3.07(0.61, 6.02) | 0.745 | 0.925 |
| HCA | 22.28(8.58, 31.49) | 19.37(9.12, 42.51) | 25.58(17.48, 73.05) | 0.086 | 0.148 |
| ACA | 2.63(1.37, 5.38) | 3.11(2.25, 5.80) | 3.91(2.23, 14.05) | 0.081 | 0.534 |
| **CA** | 89.94(42.86,228.70) | 140.00(59.23, 345.80) | 337.90(184.70,1401.00) | 0.001 | 0.018 |
| **GCDCA** | 539.00(458.4, 886.0) | 1717.00(781.1, 2749.0) | 10960.0(9831.0, 15730.0) | <0.001 | <0.001 |
| **GDCA** | 132.50(41.15,253.40) | 328.50(164.30, 484.60) | 2702.00(661.30, 4728.00) | 0.003 | 0.011 |
| **TCDCA** | 64.98(38.18, 140.90) | 192.10(106.90, 295.70) | 1100.00(720.30, 1896.00) | <0.001 | <0.001 |
| **TDCA** | 17.28(5.65, 49.36) | 62.41(28.14,118.30) | 433.50(86.58, 773.90) | 0.004 | 0.024 |
| GLCA-3S | 268.00(11.95, 429.80) | 217.20(40.66, 431.40) | 156.10(43.36, 578.30) | 0.695 | 0.860 |
| CDCA-3Gln | 11.47(7.62, 28.75) | 12.61(8.04, 19.18) | 16.79(12.82 ,29.54) | 0.337 | 0.164 |

Note: Data are represented as (Median, 25%–75% percentiles). § WT group VS HOM group. ‡ HET group VS HOM group.
